# Supplementary material for: Are carbon emissions trading and green financial instruments synergistic? -Comprehensive quantitative research based on content analysis
Source: PLoS One. 2024 Mar 7;19(3):e0298601. doi: 10.1371/journal.pone.0298601 (PMC10919601; doi:10.1371/journal.pone.0298601)
Supplement: S1 Data — (ZIP) [file pone.0298601.s001.zip › Data/地级市Z_F工作报告.zip!/║■▒▒/╥╦▓2╩╨/└┤╫╘╣1⁄2╓┌║┼í╛┬φ┐╦╩2╛▌═°í┐_2017.docx]

各位代表：

　　 现在，我代表市第五届人民政府，向大会报告工作，请予审议，并请市政协委员和列席人员提出意见。

　　一、过去五年和2016年工作回顾

　　 本届政府任期的五年，是宜昌应对严峻挑战、经受众多考验、取得重大成绩的五年。在省委、省政府和市委的正确领导下，我们深入贯彻落实党的十八大和十八届三中、四中、五中、六中全会精神和习近平总书记系列重要讲话精神，全力以赴稳增长、促改革、调结构、惠民生、防风险，完成了市五届人大历次会议确定的主要任务，实现了“十二五”圆满收官和“十三五”良好开局，为全面建成小康社会奠定了坚实基础。

　　 五年来，我们奋力赶超、争先进位，综合实力明显提升。生产总值达到3709.4亿元，是2011年的1.7倍；总量跃居全国百强城市第55位，位居中部地区同等城市第2位。地方公共财政预算收入达到300.04亿元，是2011年的2.6倍。固定资产投资达到3191.2亿元，是2011年的2.7倍。社会消费品零售总额达到1240.3亿元，是2011年的2倍。城乡居民人均可支配收入分别达到29735元、14057元，分别是2011年的1.8倍、2倍。主要经济指标年均增幅高于全国全省平均水平。宜都进入全国县域经济百强，宜都、夷陵、枝江、当阳保持全省县域经济分类考核前列。

　　 五年来，我们聚焦产业、调整结构，转型步伐明显加快。三次产业结构比例从 11.2：60.6：28.2调整为10.8：57.2：32。高新技术产业增加值达到666.2亿元，占生产总值的比重达到18%，比2011年提高8.4个百分点。规模工业总产值达到6221.8亿元，是2011年的2倍。食品饮料、先进装备制造、精细化工、现代物流、文化旅游成为千亿产业。市场主体达到30万户，是2011年的1.9倍。净增国家高新技术企业240家，达到380家。净增规模工业企业672家，达到1537家。净增产值过亿元企业418家，达到885家。9家企业产值过百亿元，3家企业进入中国企业500强，4家企业进入中国民营企业500强。国家5A级景区达到4家。进入国家重点旅游城市行列。金融机构存款余额突破3100亿元，贷款余额突破2300亿元，分别是2011年的1.8倍、2倍。新增金融机构31家，新增上市企业3家、“新三板”挂牌企业20家。农业总产值达到687亿元，其中六大特色产业产值占比超过84%，柑橘、茶叶、蔬菜、畜牧、水产综合产值均过百亿元。农产品加工产值达到2250.7亿元，与农业总产值之比提高到3.2:1。

　　 五年来，我们统筹规划、建管并重，城乡面貌明显变化。中心城区建成区拓展到190平方公里。至喜长江大桥、东山大道BRT、峡州大道等建成通车，快速路网实现内环闭合。市规划展览馆建成开放，市博物馆新馆、市奥体中心等一批大型公建项目加快建设，宜昌新区骨架基本形成。实施既有建筑综合整治项目900个。新增绿色建筑356万平方米、节能建筑2570万平方米。蝉联六届全省城乡规划建设管理“楚天杯”。预计全市常住人口城镇化率达到58.3%，比2011年提高11.3个百分点。保宜、宜巴、宜岳高速公路建成通车。实现县县通高速、通国道，乡乡通省道，村村通客车。汉宜高铁开通运营。三峡枢纽港功能提升，年吞吐量突破8000万吨。三峡机场年旅客吞吐量突破150万人次。宜昌荣获“世界可持续交通奖”。城乡网格化管理实现全覆盖，实现城区公共场所免费WiFi全覆盖和农村光纤“村村通”。五峰县城避险迁建基本完成。城乡统筹试点示范全面升级，21个镇跻身全国、全省重点镇行列，建设省级宜居村庄102个。建成高标准基本农田93万亩。

　　 五年来，我们先行先试、改革创新，内生动力明显增强。完善了宜昌高新区管理体制机制。搭建了宜昌新区、三峡旅游新区、三峡枢纽港、三峡临空经济区等发展新平台。学习对接上海自贸区，行政审批、市场准入等领域改革成效明显。“三去一降一补”取得初步成效。提前2年完成省下达的煤炭产能化解任务；商品房库存面积和去化周期进一步减少；依法妥善处置和化解一批企业风险，金融机构不良贷款率降至2.02%；降低企业成本103亿元。深化投融资制度改革，组建四大市级投融资公司，设立产业引导股权投资、绿色发展投资、城市建设等基金，7个项目进入财政部和省PPP示范项目名单，争取国际金融组织贷款5.5亿美元。不动产统一登记、农村综合产权、农村金融创新等改革顺利推进。农村土地确权登记颁证工作基本完成。农电管理体制全面理顺。“营改增”、事业单位分类、县级公立医院等改革成效明显。创新型城市、质量强市等25项国家试点示范加快推进。荣获“国家知识产权示范城市”“全国创新驱动示范市”等称号。省级以上科创平台达到188家，国家“千人计划”、省“百人计划”专家达到15人。万人发明专利拥有量达到4.7件。拥有中国驰名商标56件、国家地理标志商标36件、中国名牌产品4个、长江质量奖及提名奖5个。三峡保税物流中心封关运营。外贸出口达到23.3亿美元，是2011年的2.4倍；利用外资3.9亿美元，是2011年的1.8倍。五年实际到位招商资金5783亿元，南玻光电玻璃、惠科电子、凌云飞机维修等一批重大项目建成投产，海航通航产业园、东土科技等一批重大项目相继落户，与中省企业战略合作深入推进。实施“走出去”战略，安琪集团等48家企业到境外发展。全力服务三峡工程安全运行，争取对口支援资金537亿元。

　　 五年来，我们坚守底线、示范引领，生态建设明显进步。深入贯彻绿色发展理念，编制并实施环境总体规划，制定《城区重点绿地保护条例》，全面建立生态环保责任清单制度，进入全国首批生态文明建设先行示范区行列。关闭城区燃煤发电机组8台，淘汰燃煤锅炉335台，关停土砖窑51口，城区实现全面禁鞭，黄标车和老旧车加快淘汰，城区空气质量逐步好转。“河长制”实现全覆盖。三峡库区网箱养殖全面取缔，清江库区网箱养殖取缔工作加快推进。取缔长江干线及支流非法码头150家，关停影响环境的码头143家。治理水土流失面积332.8平方公里。完成33个中小河流治理项目。城区黑臭水体治理加快实施。单位生产总值能耗、地耗、用水量分别下降17%、30%和33%，成为“全国国土资源节约集约模范市”。全面超额完成省政府下达的节能减排任务。城区人均公园绿地面积达到14.33平方米，“六大水系、八大公园”生态景观雏形初显。创建市级以上生态村镇798个。全市森林覆盖率达到65.7%，成为“国家森林城市”。

　　 五年来，我们保障民生、促进和谐，人民福祉明显改善。民生支出占地方公共财政预算支出总额的比重保持70%以上，年均增长23.6%。32万人口脱贫销号，8015户1.9万人实现易地扶贫搬迁脱贫，12万人实现政策兜底脱贫。累计新增城镇就业57万人，转移农村劳动力就业47万人，城镇登记失业率控制在3.03%以内。五项保险参保410万人次，养老、医疗保险制度实现全覆盖。社会救助水平稳步提升。连续两年进入中国城市“慈善百强”。实施保障性安居工程23.7万套，其中棚户区改造14.9万套。改造农村危房6万余户。累计解决220万农村人口饮水安全问题。国家食品安全示范城市创建试点通过中期评估。安全生产工作持续改进。“一本三化”社会治理创新、吸毒人员网格化服务管理经验在全国推广。公众安全感和治安满意度连续五年保持全省前列，荣获全国社会管理综合治理“长安杯”。

　　 以社会主义核心价值观引领精神文明建设，蝉联两届“全国文明城市”。入选第三批创建国家公共文化服务体系示范区。被命名为“中国诗歌之城”“中国钢琴之城”。2件作品获“五个一工程”奖。全民健身运动蓬勃开展，竞技体育水平不断提高。学前三年毛入园率提高到90%。义务教育均衡发展示范县创建实现全覆盖。高中阶段教育全面普及。三峡大学实现整体一本招生，办学水平进入全国高校200强。三峡职院办学质量明显提升。市一中、夷陵中学、市委党校三个新校区基本建成，市青少年实践教育基地、妇女儿童活动中心、老年大学等重点项目加快建设。计划生育国策全面贯彻落实。城乡医疗卫生服务体系不断完善，“互联网+分级诊疗”惠民医疗服务受到国务院表彰。蝉联三届“国家卫生城市”。人均期望寿命达到77.72岁，成为首批“全国健康城市试点”。

　　 国防动员、民兵预备役建设不断加强，蝉联六届“全国双拥模范城”。广播影视、新闻出版、移民、物价、粮食、供销、统计、审计、对台、外事、侨务、港澳、民族、宗教、社科、保密、信访、档案、方志、科普、妇女、儿童、援藏、援疆等各项工作都取得新成绩，气象、防震减灾、人防、老龄、残疾人等各项事业都取得新进步。

　　 五年来，我们转变职能、强化服务，自身建设明显加强。认真贯彻中央八项规定精神，严格落实全面从严治党主体责任，深入开展党的群众路线教育实践活动、“三严三实”专题教育和“两学一做”学习教育，始终把纪律和规矩挺在前面，政府系统党风廉政建设取得新成效。地方立法工作有序推进，综合执法体制改革深入实施，政府法律顾问制度、行政机关负责人出庭应诉制度全面推行，法治政府建设进程加快。“六五”普法全面完成。认真执行市人大及其常委会的决议、决定，自觉接受市人大及其常委会法律监督和工作监督，主动接受市政协民主监督和社会舆论监督，按期办复人大代表议案和建议1059件、政协委员建议案和提案2707件。简政放权、放管结合、优化服务改革深入推进，市级政府部门权责清单制度全面建立，政府职能加快转变，行政效能持续提升。

　　 刚刚过去的2016年，我们认真践行五大发展理念，积极推进供给侧结构性改革，科学应对“98+”洪涝灾害和经济下行压力，经济社会发展稳中有进、稳中有转、稳中向好。全年生产总值增长8.8%，工业增加值增长10.2%，固定资产投资增长18.8%，社会消费品零售总额增长13.8%，城乡居民人均可支配收入分别增长9%和8.2%。三峡生态经济合作区、三峡综合交通运输体系、湖北自贸区宜昌片区等一批重大事项上升为国家战略，呼南高铁宜昌段、郑万高铁连接线等一批重大项目进入国家建设规划。三峡生态治理“宜昌试验”取得初步成效。灾后恢复重建、精准扶贫、社会救助等经验在全省推广。

　　 各位代表，五年来，全市上下风雨同舟，携手共进，展示了新常态下的新作为，取得了有目共睹的新业绩。这是中央和省委省政府坚强领导、深切关怀的结果，是市委统揽全局、科学决策的结果，是市人大市政协有效监督、大力支持的结果，是全市人民共克时艰、真抓实干的结果。在此，我谨代表市人民政府，向人大代表、政协委员，向广大干部群众、离退休老同志，向各民主党派、工商联、无党派人士和社会团体，向驻宜部队官兵、中省驻宜单位，向所有关心支持宜昌改革发展的海内外朋友，表示衷心感谢并致以崇高敬意！

　　 我们也清醒地看到，宜昌综合实力还不够强，发展的质量还有待进一步提高，城市的辐射带动作用还需进一步提升，与沿江沿海先进城市相比差距还很大；实体经济仍面临不少困难，民间投资活力不足，科技含量高、带动能力强的好项目大项目仍然不多；转型发展面临不少困难，经济结构不优，创新动能不足，新旧动能转换亟待加速，开放型经济发展水平总体不高，转型升级任重道远；绿色发展面临不少严峻挑战，生态建设、环境保护、污染治理与中央要求、与群众期盼还有较大差距，突出环境问题整治还需要进一步加大力度；安全生产形势依然严峻，安全生产领域仍然存在薄弱环节；民生改善与人民群众的期盼还有不小的差距，基本公共服务总量不足、水平不高、配置不均，贫困人口数量仍然较多，扶贫脱贫任务艰巨，城乡区域发展不平衡、不协调的问题依然存在；体制机制改革还需攻坚克难；少数干部不作为、慢作为、不会为现象依然存在，一些领域和地方腐败现象时有发生。我们将突出问题导向、突出工作重点、突出落细落小，坚决采取有效措施，努力把工作做得更好，决不辜负全市人民的期望和重托。

　　二、今后五年工作建议

　　 今后五年，是宜昌率先全面建成小康社会的决胜阶段。宜昌面临长江经济带、中部崛起、长江中游城市群、三峡后续工作规划和省“一元多层次”战略等重大发展机遇，具备产业基础扎实、开放优势明显、发展空间广阔等转型跨越的有利条件。只要我们适应把握引领经济发展新常态，始终保持战略定力，坚持把加快发展作为第一要务、把改革创新作为根本动力、把民生幸福作为不懈追求，团结拼搏、务实重行、攻坚克难，就一定能推动宜昌发展再上新台阶、实现新跨越、铸就新辉煌！

　　 按照市第六次党代会的安排部署，今后五年我们将全面贯彻党的十八大和十八届三中、四中、五中、六中全会精神，深入贯彻习近平总书记系列重要讲话精神，围绕“五位一体”总体布局和“四个全面”战略布局宜昌实施，全面落实五大发展理念，坚决落实“共抓大保护、不搞大开发”重要精神，推进绿色发展，促进转型跨越，加快建设综合实力更强的省域副中心城市、全国区域性中心城市和世界水电旅游名城，加快建设宜居、宜业、宜旅、宜学、宜养的宜人之城，确保在全省率先实现精准脱贫、率先全面建成小康社会，综合实力进入中西部和长江沿线同等城市前列、进入全国城市50强，加快建设“强、大、优、美”现代化特大城市，奋力谱写全面建成小康社会新篇章。

　　 今后五年经济社会发展主要预期目标是：

　　生产总值年均增长8.5%左右，固定资产投资年均增长16%左右，社会消费品零售总额年均增长12%左右，外贸出口年均增长5%左右，城乡居民收入增长与经济增长基本同步。

　　 今后五年的工作思路是：

　　 （一）坚定不移推进创新发展。深入实施创新驱动战略，加快建设国家创新型城市、国家知识产权强市、创新创业示范城市。建立健全创新激励机制，力争全社会研发投入占生产总值比重超过2.5%，每万名从业人员中研发人员数量、每万人发明专利拥有量均翻一番。深入实施高质量可持续发展战略，建成全国质量强市示范城市。积极发展新技术、新产业、新业态、新模式“四新”经济，加快新旧动能转换，推动产业向中高端水平迈进。发展壮大先进装备制造、新材料、生物医药、新一代信息技术、节能环保与新能源、食品饮料、精细化工、建筑、文化旅游、现代物流等千亿产业，着力培育民用航空航天、康养等新兴产业，把实体经济做得更实、更强、更大、更优。力争高新技术产业和现代服务业增加值占生产总值比重年均提高1个百分点以上。

　　 （二）坚定不移推进协调发展。坚持区域协同、城乡一体、两个文明并重、军民融合发展。实施城乡全域规划，完善“一主六副多层次”城镇体系，形成大、中、小城市和小城镇协调发展新格局。推动宜昌高新区、宜昌新区、三峡枢纽港、三峡旅游新区、三峡临空经济区产城融合、协同发展。加快推进以人为核心的新型城镇化，力争全市常住人口、户籍人口城镇化率分别提高7个和8.5个百分点。坚持“双轮驱动”，实现城区经济与县域经济协调并进。推动城乡空间布局联动优化、基础设施联动建设、产业园区联动开发、经济结构联动升级、社会事业联动发展。以社会主义核心价值观为引领，厚植“诗歌之城”“钢琴之城”“读书之城”文化优势，加快打造“全国文明城市群”。完善军民融合发展体制机制，打造全国重要的军民融合特色产业基地。（三）坚定不移推进绿色发展。始终坚持节约资源和保护环境的基本国策，牢固树立“绿水青山就是金山银山”的强烈意识，坚决把修复长江生态环境摆在压倒性位置，坚定走生产发展、生活富裕、生态良好的文明发展道路。认真落实主体功能区规划，促进生产空间集约高效、生活空间宜居适度、生态空间山清水秀。实行最严格的环境保护制度，深入实施大气、水、土壤污染防治行动计划，全面推进绿色低碳循环发展。推进绿色城市、海绵城市建设，大力发展绿色建筑，加快棚户区改造和城市整理。创建国家新型智慧城市示范城市。完善绿色发展体制机制，改革生态环境保护绩效考评制度，全面实行领导干部自然资源资产离任审计。全面落实生态文明制度，加快形成绿色发展方式和绿色生活方式，加快建设国家生态文明建设先行区和全国绿色发展示范城市，努力为全市人民提供更多的绿色福利。（四）坚定不移推进开放发展。深入实施开放先导战略，全面提升开放型经济水平，完善高水平双向开放体制机制，持续优化开放发展环境，建成更具活力和竞争力的内陆开放高地。系统推进三峡生态经济合作区、三峡综合交通运输体系、湖北自贸试验区宜昌片区、“宜新欧”国际物流新通道和宜昌综合保税区建设，打造改革开放新引擎。积极推进大通关建设，完善提升航空口岸和水运口岸功能。完善铁、水、公、空、管立体化交通格局，打造全国重要的区域性综合交通枢纽。健全“引进来、走出去”协同推进机制，加强与长三角、京津冀、珠三角、成渝城市群、三峡对口支援省市的区域合作，深化与中省企业、科研院所、高等院校的战略合作，推动资本、技术、人才等深度对接、互利共赢。（五）坚定不移推进共享发展。坚持以人民为中心的发展思想，高度重视、认真解决人民群众普遍关心的突出问题，不断增强人民群众的获得感。坚决打赢脱贫攻坚战，确保在全省率先全面实现贫困人口销号、贫困村出列、贫困县摘帽。全面发展社会事业，不断完善社保体系，提供更充分、更均等的基本公共服务，促进社会公平正义，实现全市人民共同迈入全面小康社会。加快建设区域性医疗卫生中心，建成全国健康城市。创建国家级三峡康养试验区。加快建设以国家食品安全示范城市为引领的“食品安全城市群”。牢固树立安全发展理念，不断提升安全生产总体水平，确保人民群众安康幸福。构建全民共建共享的社会治理格局，提高社会治理能力和水平，实现社会充满活力、安定和谐。

　　三、2017年工作重点

　　 今年是实施“十三五”规划的重要一年，是供给侧结构性改革的深化之年，做好今年工作、实现新一届政府工作良好开局，意义重大。全市经济社会发展主要预期目标是：生产总值增长8.5%左右，规模工业增加值增长9.5%左右，固定资产投资增长16%左右，社会消费品零售总额增长12.5%左右，外贸出口总额增长4%左右，城乡居民收入增长与经济增长基本同步，城镇登记失业率控制在4%以内，全面完成省政府下达的节能减排和环境保护任务，促进经济平稳健康发展和社会和谐稳定，以优异成绩迎接党的十九大胜利召开。

　　 围绕实现上述目标，我们将坚持稳中求进工作总基调，以提高发展质效为中心，以推进供给侧结构性改革为主线，重点做好以下六个方面工作。

　　 （一）深化改革开放，实施创新驱动，增创跨越发展新优势

　　 更大力度推进改革。以简政放权、放管结合、优化服务改革为突破口，进一步精简行政审批事项，加强事中事后监管，清理规范前置审批和中介服务事项，提高行政审批效率。完善企业登记“五证合一”、个体工商户登记“两证整合”制度。加快社会信用体系建设，健全失信惩戒机制。推进“互联网+政务服务”，确保基本公共服务事项80%以上可在网上办理。深化国企国资改革，积极稳妥发展混合所有制经济，支持国企投资项目引入非国有资本，支持中省在宜国企“三供一业”分离移交工作，确保国有资产保值增值。深化财税金融体制改革，加强政府性债务管理，提高预算管理绩效，推动金融监管体制改革。加快综合执法改革和“互联网+分级诊疗”惠民医疗服务改革。扎实推进农业供给侧结构性改革，推进农村承包土地“三权分置”改革。统筹抓好文化、公安、统计、国有林场等各项改革。

　　 持续推进创新创业。加快实施“科技型中小企业创新成长工程”“高新技术企业绩效倍增工程”，确保新增国家高新技术企业40家以上，高新技术产业增加值增长15%以上。加快科创平台建设，确保新增省级以上技术研发平台2家以上，新建成孵化器面积10万平方米以上，在孵企业达到600家以上。完成科技成果转化扩散项目100项以上。大力发展天使投资、创业投资等风险投资机构。推动设立科技银行。加强知识产权保护，力争发明专利授权量突破500件。确保通过国家创新型试点城市验收。实施精品名牌战略，推行卓越绩效管理，力争新增长江质量奖或提名奖1个、中国驰名商标5件。完善人才引进、培养、使用、激励机制，统筹推进各类人才队伍建设，做强人才保障支撑。

　　 提升对外开放水平。扎实推进湖北自贸区宜昌片区建设。加快引进一批外贸出口企业进驻三峡保税物流中心。抓好综合保税区申报建设。抓紧编制三峡临空经济区产业发展、空间布局等规划。加快三峡机场二期改扩建、凌云飞机维修基地喷涂等项目建设。筹建三峡航空公司。力争新开通2条国际旅游包机航线。拓宽利用外资领域和渠道，力争实际利用外资增长10%。扩大生物医药、装备制造、农产品等出口优势。精心举办“一带一路·中国宜昌”茶叶、柑桔国际采购商大会。深化与长江经济带、三峡生态经济合作区城市交流合作，积极参与区域通关一体化和检验检疫一体化改革。

　　 （二）加快产业发展，做强实体经济，培育转型升级新动能

　　 积极扩大有效投资。充分发挥投资对稳增长的“定海神针”作用，突出抓好产业、民生、基础设施三大类投资，确保投资总规模突破3600亿元。完善重大项目推进机制，加快海航通航产业园、兰台科技园、东土科技等项目建设，推动贝因美婴童产业园、华环迪拉汽车排气系统智能制造等项目尽快开工，促进100个省市重点项目早建成、早见效。加大项目前期谋划和推进力度，争取更多项目进入国家和省投资规划。推动一批PPP入库项目早日落地。力争债券融资突破50亿元。坚持“房子是用来住的、不是用来炒的”定位，促进房地产市场平稳健康发展。支持建筑业转型升级，力争产值突破1000亿元。强化产业招商、以商招商、驻点招商、海外招商、联动招商，重点引进电子信息类龙头企业，突破性引进临空产业、医疗器械、高端装备制造类项目。力争招商引资实际到位资金1500亿元，新开工亿元以上招商项目380个。深化与中省企业战略合作，推动一批项目尽快落地。发挥三峡对口支援和经济协作等平台优势，确保到位资金110亿元。

　　 推动工业提质增效。深入推进“双五双百”工程，壮大食品饮料、先进装备制造等支柱产业，力争产值分别达到1900亿元、1330亿元；加快发展新材料、生物医药、新一代信息技术、节能环保与新能源等新兴产业，力争产值分别达到910亿元、550亿元、380亿元、500亿元。培育民用航空航天、海洋工程、磁电子等军民融合产业。调整化工产业布局，推动化工产业向精细化、绿色化转型。实施华强科技等218个技改项目。选择60家企业开展“两化”融合对标试点，新建一批智能工厂和车间。抓好80个重点工业项目建设的监控考核。实施“小进规”三年滚动计划，确保今年新增规模工业企业110家以上。力争规模工业总产值突破6800亿元。加大“去产能”力度，化解行业过剩产能，坚决淘汰落后产能。稳步推进困难企业重组解困。依法处置“僵尸企业”。从税费、融资、物流、用工、制度性交易等方面着手，千方百计为市场主体“降成本”。推进用电大户直接交易试点扩容，争取售电侧改革试点。

　　 加快发展现代服务业。实施千亿文化旅游产业三年行动计划，力争接待游客6500万人次，旅游总收入达到700亿元。以三峡旅游新区为重点，加快平湖半岛、三峡游轮中心、南津关小镇等项目建设，推动宜昌中华恐龙园、三峡旅游咨询服务中心等项目尽快开工，确保三峡国际房车露营地建成运营。支持远安、夷陵、秭归、长阳、五峰创建国家全域旅游示范区，西陵创建省级全域旅游示范区。支持三峡大瀑布创建国家5A级景区。加快三峡香溪、百里荒、大老岭、枝江金湖等重点旅游项目建设。推进12条生态旅游景观廊道建设。创建一批特色旅游名镇、旅游名村。加快发展文化创意产业，力争新增2个省级以上文化产业示范园区。加快三峡翻坝物流园、白洋物流园和13个县级电商园建设，建成一批区域性仓储物流配送基地，力争现代物流业产值达到1400亿元。抓好传统“老字号”创建和传承，促进实体零售商业企业创新升级。确保新增限额以上商贸企业100家。制定康养产业规划，加快清江康养试验区、江南康养城项目建设，支持三峡职院附属医院建设康养护理人才培训示范基地，争取第四届中国康养产业发展论坛在宜举办。积极引进金融机构落户宜昌，支持地方法人金融机构做大做强。推动更多优质企业上市、挂牌。依法严厉打击非法集资、金融欺诈等行为，有效防范和化解金融风险，进一步优化金融生态环境。

　　 提升现代特色农业竞争力。以农业供给侧结构性改革为契机，狠抓农产品标准化生产、品牌创建、质量安全监管，不断增加绿色优质农产品供给，促进农业提质增效、农民持续增收。培育“宜昌蜜桔”“秭归脐橙”“清江椪柑”等精品名果。打造“宜昌绿茶”“宜昌红茶”“湖北青砖茶”区域公共品牌。积极发展优质畜牧业。壮大水产、食用油产业规模，加快发展花卉苗木、中药材、食用菌产业。大力发展农产品精深加工，力争农产品加工产值突破2300亿元。积极培育农村电商、农产品定制等“互联网+”新业态。加快建设国家农业科技园、国家柑桔公园、三峡茶谷、宜红茶博览园。大力培育新型经营主体和服务主体，引导农民规范发展多种形式的适度规模经营。强化农产品产地准出监管，创建一批省级以上农产品质量安全县（市）。建成高标准基本农田40万亩。实施农业抗旱能力提升三年行动计划。加快新一轮农村电网改造升级。开展第三次农业普查。

　　 （三）强化城乡统筹，注重协调联动，塑造城市发展新品质

　　 加快城市新区建设。坚持人民城市为人民，走内涵式、集约型、生态化的城市发展之路，让人民群众在城市生活得更方便、更舒心、更美好。加快建设快速路网，建成通车道路60公里，基本形成城市外环骨架。加快伍家岗长江大桥、西陵二路、峡州大道二期、点军大道二期等项目建设，启动江城大道二期、花溪路二期、胜利三路和港窑路延伸段等项目建设，确保BRT夜明珠段上半年建成通车。加快大剧院（音乐厅）、科技馆、档案馆等功能性项目建设，确保市博物馆新馆、奥体中心体育馆、市一中新校区、夷陵中学新校区、市委党校新校区等投入使用。加快推进滨江中央商务区、唐家湾片区、奥体中心片区、卷桥河片区等综合开发。实施将军路、江南二路等地下综合管廊建设。加快建设海绵城市样板区。确保新区建设投资完成400亿元以上。

　　 加强城市精细管理。完成城市总体规划修编。继续抓好光纤入户、无线网络全覆盖、县市城区公共场所免费WiFi全覆盖等工程。加快智慧城管建设，力争年内50%县市建成数字城管。加快建设城区智能交通，完善交通微循环系统，努力缓解交通拥堵。加快城区公共自行车系统一期项目建设。加强城区公共停车场建设，新增公共停车泊位2500个。推广生活垃圾分类，建立餐厨垃圾收运和执法体系。强化市容环境综合治理。完善违法建筑快查快处机制。加强城市防洪排涝设施建设。完成250栋既有建筑综合整治。推进城区老旧小区综合治理。继续实施城市夜景灯光提升工程。提高住宅小区物业规范化管理水平。

　　 完善城乡交通网络。推进重大交通项目建设，确保完成交通投资130亿元。开工建设宜张高速五峰至湘鄂界段，加快建设江北翻坝高速公路。开工建设点军联棚至长阳龙舟坪等7条一级公路，加快建设远安盘古至棚镇等6条一级公路。抓好点军、宜都、五峰、远安客运站建设。加快城乡道路客运班线公交化改造。力争实现城际公交线路全覆盖。新建农村公路1000公里。加强乡村道路维护与管理。加快香溪长江大桥、白洋长江大桥建设，力争长江溪大桥开工建设。续建云池二期、三峡翻坝转运中心码头等6个项目，启动白洋港二期、香溪河航道整治等5个项目，确保白洋港一期、茅坪港二期等建成营运。加快推进呼南高铁宜昌段、郑万高铁连接线、三峡铁水联运等铁路项目前期工作。积极争取城市轨道交通规划获批。抓好宜昌东至小溪塔既有铁路改造。加快远安至当阳地方铁路建设。

　　 优化区域发展格局。鼓励各县市区和宜昌高新区因地制宜，创新发展，竞进提质。加快宜昌高新区园区基础设施建设，新建一批标准厂房，确保新引进项目80个以上，完成投资200亿元。支持西陵区建设现代服务业示范区、伍家岗区建设都市经济样板区、点军区创建绿色发展示范区、猇亭区建设产业发展核心区、夷陵区建设城乡统筹示范区。支持宜都、夷陵、枝江、当阳、远安争先进位，支持长阳、秭归、五峰、兴山脱贫攻坚。支持山区县、库区县到沿江地区发展“飞地经济”。支持宜都、长阳开展国家新型城镇化试点。加快建设11个新型城镇化试点镇、4个城乡统筹试验示范区、9条城乡统筹示范带。

　　 （四）突出绿色发展，狠抓环境治理，构建美丽宜昌新生态

　　 强力推进污染防治。加大机动车和船舶尾气、工业废气、燃煤、建筑扬尘、餐饮油烟、秸秆露天焚烧等防治力度，确保全年空气质量优良天数比率超过72%。深化“河长制”改革。严格集中式饮用水水源地保护。强化长江宜昌段干支流综合治理。加大清江、黄柏河、沮漳河、香溪河等流域污染防治力度，确保考核断面水质达标。完成清江库区网箱养殖取缔任务。加快整治城区11条黑臭水体。强化排污口和涉磷企业监管。巩固长江及其支流岸线治理成果，完成“规范一批、提升一批”码头治理工作。完成沙河、临江溪、点军第一污水处理厂扩容提标工程，加快点军第二污水处理厂建设。开展土壤污染状况调查，建立建设用地土壤环境质量评估制度，全面整治矿山尾矿、工业固体废弃物堆存场所。强化环境质量网格化监控。推动重点企业、重点单位污染物排放在线监控全覆盖。推行环境污染第三方治理。加强环境应急能力建设。坚决抓好突出环境问题的整改，确保认识到位、措施到位、整改到位。对环境违法行为零容忍，形成督查问责威慑力。严格执行生态环保责任清单制度，坚决落实“党政同责、一岗双责、失职问责、终身追责”。加强生态文明宣传教育，增强全民生态环保意识。

　　 积极开展生态修复。深入推进生态治理“宜昌试验”，抓好生态守护、生态产业、生态金融、生态公民等试点工作。加强三峡库区生态保护和治理。加快磨基山公园、求雨台公园、卷桥河湿地公园、柏临河湿地公园等建设，启动东山公园二期、松林公园、石板森林公园等建设。加快城区山体生态修复，全面启动建成区山体绿化工程。完成植树造林4.9万亩、封山育林16万亩。积极发展装配式建筑。新增绿色建筑面积110万平方米。加快建设农村清洁能源、生态家园。新建25个美丽宜居村庄。治理水土流失面积300平方公里。启动全市第三次污染源普查。全面实施控制污染物排放许可制。抓好自然资源资产负债表编制改革试点和领导干部自然资源资产离任审计。

　　 节约集约利用资源。严格落实资源环境生态红线保护制度。积极实行能源和水资源消耗、建设用地等总量和强度双控行动，推动能耗、地耗、水耗强度持续下降，加快创建国家节水型城市。推进农业废弃物资源化利用，创建一批生态循环高效农业示范县、示范乡。实施重点行业清洁生产提升计划，推动工业绿色转型。深入推进猇亭园区、枝江等国家循环经济试点建设，支持夷陵创建国家资源综合利用示范基地。加大闲置和低效利用土地清理处置力度，巩固“全国国土资源节约集约示范市”创建成果。

　　 （五）坚持共建共享，努力改善民生，顺应人民群众新期盼

　　 扎实推进精准扶贫。突出“准、实”导向，集中力量打好产业扶贫提质、搬迁扶贫提能、教育扶贫提智、兜底扶贫提标、生态扶贫提升五大战役。因地制宜培育主导产业，支持各类新型经营主体与贫困村、贫困户建立利益联结机制，带动贫困对象稳定增收。抓好光伏扶贫。加快贫困地区基础设施建设。强化对扶贫资金的监管。实现1.1万户3.2万人易地扶贫搬迁脱贫。确保12万贫困人口销号、92个贫困村出列、远安县脱贫摘帽。

　　 加强社保体系建设。扎实抓好重点群体就业工作，支持农村劳动力转移就业和农民工返乡创业。确保城镇新增就业7.6万人。加快实施全民参保计划，确保五项保险参保达到624万人次。整合城乡居民基本医疗保险制度，深化复合式医保付费方式改革。稳步提高社会救助、优抚安置、社会福利等社会保障水平。启动亚行贷款综合养老示范项目建设。深入推进国家食品安全示范城市创建，确保通过国家考核验收。实施棚户区改造2.3万套。改造农村危房1.2万户。实施农村50万人饮水安全巩固提升工程。落实粮食安全行政首长负责制。组建宜粮集团。抓好水库移民后期扶持政策落实，推动移民安稳致富。

　　 大力发展社会事业。深入推进社会主义核心价值观建设，巩固提升文明城市创建成果，确保蝉联“全国文明城市”。支持宜都创建“全国文明城市”。加快创建国家公共文化服务体系示范区。推出一批文艺精品力作。加快城乡公共体育设施建设。办好中国长江三峡国际旅游节、宜昌长江钢琴音乐节、市第二届艺术节、市第五届运动会等节赛活动。完成二期学前教育三年行动计划。巩固义务教育均衡发展和示范县创建成果。实施高中阶段教育结构性改革。开工建设市特校新校区。力争三峡航空学院申办成功。支持三峡职院、三峡电力职院、三峡旅游职院加快发展。支持三峡大学建设一流大学、一流学科。全面推进“健康宜昌”建设，抓好健康城市国家试点。加快推进市妇女儿童医院、市中医院门诊急诊综合大楼等项目建设。实施第二轮乡村医生定向委托培养工程。实施全面两孩政策，促进人口均衡发展。加强外事、侨务、港澳、对台、保密工作。继续抓好档案、方志、供销、科普、社科、慈善、人防、消防、气象、援藏、援疆等各项工作。

　　 着力打造平安宜昌。完善城市社区“一本三化”和农村“三化四务”服务体系，全面提升城乡社会治理社会化、法治化、智能化、专业化水平。推进村（居）民自治和社区协商民主。加快建设全国公共安全视频监控联网应用示范城市，继续抓好吸毒人员网格化服务管理试点，构建立体化治安防控新格局。牢固树立安全生产红线意识，坚持安全第一、预防为主、综合治理，严格落实安全生产责任制，以铁的手腕、铁的纪律、铁的作风，抓好安全生产隐患排查和整改，坚决遏制重特大安全生产事故发生。全面实施“七五”普法规划。加强人民调解、行政调解、司法调解、法律援助等工作。加强和改进信访工作。支持群团组织参与社会治理和服务经济发展。加强应急管理和防灾减灾体系建设。支持军队改革。深化全民国防教育，增强全民国防意识。巩固“全国双拥模范城”创建成果。推动民族团结进步。依法管理宗教事务。

　　 （六）全面从严治政，加强自身建设，开创政府工作新局面

　　 依法行政，接受监督。把政府工作全面纳入法治轨道，依法全面履行政府职能，不断提高政府工作人员法治思维和依法行政能力，加快建设法治政府。强化行政复议和应诉监督，促进严格规范公正文明执法。自觉接受人大及其常委会法律监督、工作监督和政协民主监督，认真办好议案提案。加强与各民主党派、工商联、无党派人士和人民团体联系，广泛听取各方面意见建议。全面推进政务公开，增强决策透明度。积极运用全媒体手段，加强政策宣传解读，让群众看得到、听得懂。强化政务舆情收集研判，积极回应社会关切。

　　 务实重行，提升效能。坚持立标杆、作表率，少说多干、真抓实干、埋头苦干，以“工匠精神”推动各项工作落实落地，以创新精神破解制约地方发展的矛盾和问题，努力干出新业绩、开创新局面。坚持开短会、发短文、讲短话，集中精力谋大事、抓实事、解难事。坚持言必信、行必果，认准的事情一抓到底，承诺的事项坚决兑现，切实做到雷厉风行、快捷高效。加强精准督查，确保中央精神全面贯彻落实，确保省委、省政府和市委决策部署落地生根，确保政令畅通、令行禁止。

　　 遵规守纪，履职尽责。牢固树立政治意识、大局意识、核心意识、看齐意识，严格落实全面从严治党主体责任，切实加强党风廉政建设和反腐败斗争。深入落实中央八项规定精神，驰而不息整治“四风”。加强行政监察和审计监督，坚决纠正损害群众利益的不正之风，以零容忍态度惩治腐败行为。全力支持纪检监察机关履行监督责任。加大行政问责和领导干部问责追责力度，坚决纠正行政不作为、乱作为，坚决查处领导干部履职不尽责、不到位行为，坚决惩处失职、渎职行为。

　　 各位代表，蓝图已绘就，扬帆正当时。让我们紧密团结在以习近平同志为核心的党中央周围，在省委、省政府和市委的坚强领导下，潜心静气，埋头苦干，务实重行，克难奋进，全力推进绿色发展、促进转型跨越，加快实现“两个率先、两个进位”奋斗目标，奋力谱写全面建成小康社会新篇章！
